# *Chlamydia trachomatis* growth and development requires the activity of host Long-chain Acyl-CoA Synthetases (ACSLs)

**DOI:** 10.1038/srep23148

**Published:** 2016-03-18

**Authors:** Maria A. Recuero-Checa, Manu Sharma, Constance Lau, Paul A. Watkins, Charlotte A. Gaydos, Deborah Dean

**Affiliations:** 1 Center for Immunobiology and Vaccine Development, UCSF Benioff Children’s Hospital Oakland Research Institute, Oakland, CA, 94609, USA; 2 Department of Infectious Disease, Johns Hopkins University, Baltimore, MD, 21205, USA; 3 Hugo W. Moser Research Institute at Kennedy Krieger, Baltimore, MD, 21205, USA; 4 Department of Neurology, Johns Hopkins University School of Medicine, Baltimore, MD, 21205, USA; 5 Department of Bioengineering, University of California at Berkeley and San Francisco, CA, USA

## Abstract

The obligate-intracellular pathogen *Chlamydia trachomatis* (*Ct)* has undergone considerable genome reduction with consequent dependence on host biosynthetic pathways, metabolites and enzymes. Long-chain acyl-CoA synthetases (ACSLs) are key host-cell enzymes that convert fatty acids (FA) into acyl-CoA for use in metabolic pathways. Here, we show that the complete host ACSL family [ACSL1 and ACSL3–6] translocates into the *Ct* membrane-bound vacuole, termed inclusion, and remains associated with membranes of metabolically active forms of *Ct* throughout development. We discovered that three different pharmacologic inhibitors of ACSL activity independently impede *Ct* growth in a dose-dependent fashion. Using an FA competition assay, host ACSLs were found to activate *Ct* branched-chain FAs, suggesting that one function of the ACSLs is to activate *Ct* FAs and host FAs (recruited from the cytoplasm) within the inclusion. Because the ACSL inhibitors can deplete lipid droplets (LD), we used a cell line where LD synthesis was switched off to evaluate whether LD deficiency affects *Ct* growth. In these cells, we found no effect on growth or on translocation of ACSLs into the inclusion. Our findings support an essential role for ACSL activation of host-cell and bacterial FAs within the inclusion to promote *Ct* growth and development, independent of LDs.

*Chlamydia trachomatis* (*Ct*) is an obligate intracellular Gram-negative pathogen that causes a wide range of human diseases involving the eye, and urogenital and respiratory tracts. *Ct* represents a pressing global public health burden since it is the leading cause of preventable blindness and bacterial sexually transmitted diseases in the world today^1^.

*Ct* actively modulates its lipid composition both at the inclusion and the bacterial membranes within hours of entry into the host cell and during replication. A growing body of evidence shows that *Ct* recruits into the inclusion different pools of host-derived lipids, such as ceramide, sphingomyelin^2^,^3^,^4^,^5^,^6^,^7^, cholesterol^8^, cardiolipin^9^, and phosphatidylcholine^9^,^10^. More recent studies suggest that, although *Ct* is able to synthetize the lipids required for its membrane systems without the need for host phospholipids^11^, the bacteria are still able to hijack host-lipid pathways to obtain host fatty acids (FA)^12^. The bacteria also recruit into the inclusion host enzymes that are involved in lipid trafficking and biosynthesis, such as the ceramide transfer protein (CERT) and high-density lipoprotein (HDL) biogenesis machinery^4^,^13^,^14^. *Ct* intercepts multiple trafficking pathways in the host cell to incorporate these essential metabolites and enzymes for its survival^15^. One of the proposed mechanisms is via lipid droplets (LD), which are lipid storage organelles that are present in all eukaryotic cells. Some studies have reported the recruitment of LDs into the *Ct* inclusion and the modification of host LDs in response to *Ct* infection^16^,^17^,^18^,^19^.

Host lipid biosynthesis is directly dependent on acyl-CoA synthetases, a family of isozymes that activate FAs, derived from either external or internal cellular sources, to produce acyl-CoA. Acyl-CoA is an essential metabolite that is rerouted to different lipid synthesis and/or degradation pathways to obtain energy, depending on cellular needs^20^.

Long-chain acyl-CoA synthetases (ACSLs) are a subfamily of five isozymes (ACSL1, ACSL3, ACSL4, ACSL5 and ACSL6) present in different tissues and organs. ACSLs convert long-chain FAs with acyl chains ranging from C12 to C18 into long-chain acyl-CoA^21^,^22^,^23^, a necessary step for FAs to be incorporated into phospholipids. In mammals, the predominant long-chain FAs are those of 16 and 18 carbons with varying degrees of saturation^20^. Oleic acid (C_18:1_) (OA), an unsaturated long-chain FA, is commonly present in the sn-2 position of eukaryotic phospholipids^9^. It has previously been shown that there is an increase in long-chain FA uptake in *Ct* infected cells compared to uninfected cells, suggesting that these FAs could be beneficial for *Ct* growth^24^. Recently, it has been shown that *Ct* is able to incorporate host long-chain FAs into the bacterial phospholipids, with a preference for saturated FAs. However, 8% of the FAs present in *Ct* phospholipids are OA, which is not synthetized by *Ct*^12^. *Ct* is able to synthetize both straight and branched-chain saturated FAs, with the most abundant branched-chain FAs being ante-iso and iso C15:0 9.

Several publications have shown that ACSLs are important for the development of some pathogens, such as cytomegalovirus and picornavirus^25^,^26^. ACSL3 has been identified as a novel host factor required for picornavirus replication. A rapid increase in long-chain FA import into picornavirus-infected cells has been linked to activation of acyl-CoA synthetase. These incorporated FAs are used for phosphatidylcholine synthesis while, in uninfected cells, they are stored in LDs. These data indicate that, during replication, the virus hijacks the host-cell pathways for new membrane formation.

In the present study, we show that the entire family of ACSLs is recruited into the *Ct* inclusion early in infection and that the activity of the ACSLs is essential for *Ct* development. The pharmacologic inhibition of ACSL activity, rather than the lack of LDs, is responsible for arresting *Ct* growth. Moreover, we discovered that host ACSLs are able to activate branched-chain FAs of *Ct* origin, indicating an important role for host ACSLs in the chlamydial inclusion.

## Results

### ACSLs are translocated into the *C. trachomatis* (*Ct*) inclusion during infection

**Figure 1:**
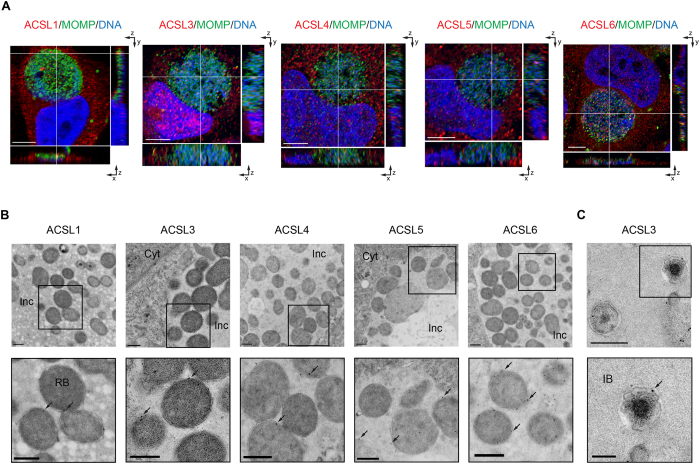
ACSLs are translocated into the *C. trachomatis* (*Ct*) inclusion during infection. (**A**) HeLa cells were infected with *Ct* L_2_ and fixed at 24 hpi. The inclusion was labeled with anti- *Ct* MOMP antibody (green), ACSL-specific antibodies (red), and Hoechst for nuclear and bacterial DNA (blue) (see Methods). Representative images of z-stack projections from confocal microscopy are shown. White lines indicate localization of the ACSLs inside the inclusion in the three planes, x, y, and z. Scale bar, 5 μm; (**B**) HEp2 cells were infected with *Ct* L_2_ for 24 h, and prepared for TEM. ACSLs were labeled with specific primary antibodies and secondary antibodies conjugated to 12 or 18 nm gold particles (see Methods). The *Ct* inclusion is shown in the upper panel and an inset at higher magnification in the lower panel. Arrows indicate the gold immunolabeling of respective ACSLs. Inc, inclusion; Cyt, cytoplasm. Scale bar, 500 nm; (**C**) HEp2 cells were infected with *Ct* L_2_ for 24 h and labeled with an anti-ACSL3 antibody as above. A portion of the *Ct* inclusion is shown on the top panel (scale bar, 500 nm) and a higher magnification inset on the bottom panel. IB, intermediate body. Scale bar, 200 nm.

To ascertain the role of the members of the ACSL family in *Ct* L_2_ infected cells, we first examined their location throughout the development of the organism. Previously, we showed that ACSL3 was recruited into the lumen of the *Ct* inclusion at 36 hours post infection (hpi)^27^. In the present study, we analyzed different time points of infection and found that ACSL1, ACSL3, ACSL4, ACSL5 and ACSL6 were all recruited into the lumen of the *Ct* inclusion as early as 6 hpi (Supplementary Fig. S1) and as late as 24 hpi (Fig. 1A). Since the inclusion size at 6 hpi is minute, we used a multiplicity of infection (MOI) of one but also a higher MOI of 50 to better visualize the inclusion and presence of ACSLs.

Using Transmission Electron Microscopy (TEM), the ACSLs were found to be localized specifically to the membranes of the metabolically active forms of the organism, reticulate bodies (RBs) and intermediate bodies (IBs), that reside in the lumen of the inclusion (Fig. 1B,C). None of the proteins were associated with other parts of the inclusion, including the inclusion membrane. The ACSLs were inside the inclusion throughout chlamydial development and were present in the inclusion of every infected cell that was analyzed by confocal and/or TEM. While a recent study claims that the fixation process can cause an engulfment of material from the host cell cytoplasm into the chlamydial inclusion^28^, our findings that the ACSL enzymes are bound to RBs and IBs and not randomly distributed inside the inclusion suggest that our data are not a result of fixation artifact. Antibodies against human cytokeratin 18 and chlamydial HSP60 were used as controls for the secondary antibodies since they are representative proteins found exclusively in the host-cell cytoplasm (cytokeratin 18) or inside the chlamydial inclusion (HSP60) (Supplementary Fig. S2). The specificity of the ACSL antibodies was determined using ACSL-specific siRNA transfection with imaging analysis by confocal microscopy (Supplementary Fig. S3A) and Western Blot (WB) (Supplementary Fig. S3B).

### ACSL activity is required for *C. trachomatis* (*Ct*) growth and development

**Figure 2:**
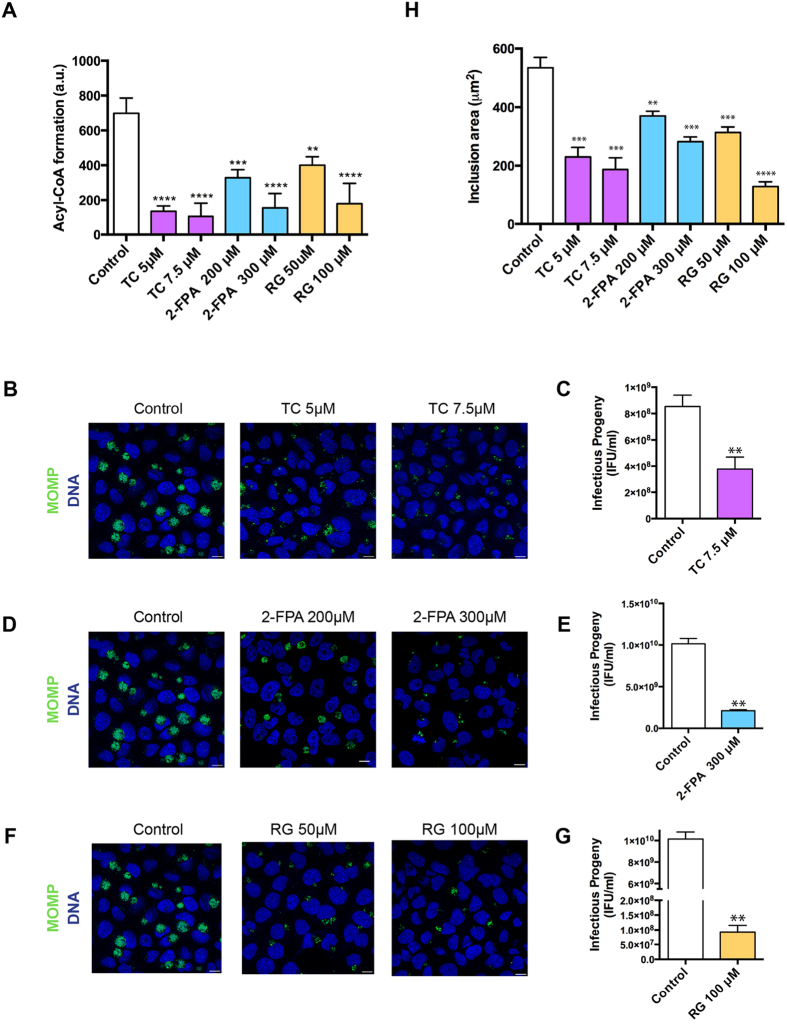
ACSL activity is required for *C. trachomatis* (*Ct*) growth and development. (**A**) HeLa cells were treated for 16 h with the following ACSL activity inhibitors: 5 μM and 7.5 μM Triacsin C (TC); 200 μM and 300 μM 2-Fluoropalmitic acid (2-FPA), and 50 μM and 100 μM Rosiglitazone (RG). The cells were lysed, and ACSL activity was measured as fluorescent acyl-CoA recovered. Error bars indicate standard deviation for three independent experiments. The asterisks indicate statistically significant differences at ^∗∗^p < 0.01, ^∗∗∗^p < 0.001, ^∗∗∗∗^p < 0.0001 by the two-tailed t-test; (**B**) HeLa cells were treated with the inhibitor TC at the indicated concentrations for 16 h and then infected with *Ct* L_2_, without removing the inhibitor from the media. At 24 hpi, the cells were fixed and prepared for confocal microscopy. Labeling was done with anti-*Ct* MOMP antibody (green) and with Hoechst to stain for nuclear and bacterial DNA (blue). Scale bar, 10 μm; (**C**) HeLa cells were treated with 7.5 μM TC for 16 h and infected with *Ct* L_2_ for 24 h, without removing the inhibitor from the media. The cultures were used for reinfecting new HeLa cell monolayers and analyzed for infectivity and production of progeny. Values (mean ± standard error for three independent experiments) are shown as inclusion forming units (IFU)/mL. Asterisks denote statistically significant differences at ^∗∗^p < 0.01, by the two-tailed t-test. The same experiment was carried out with the inhibitor 2-FPA (**D**,**E**) and RG (**F**,**G**) at noted concentrations; (**H**) HeLa cells treated with the inhibitors and infected with *Ct* as described previously were stained with anti-*Ct* MOMP antibody and imaged using confocal microscopy. The images were analyzed using Imaris X64 software to identify Regions Of Interest (ROI) coinciding with the inclusions. The software was used to calculate the mean inclusion area (in μm^2^) based on the ROIs. Values (mean ± standard error for three independent experiments) are shown. The asterisks indicate statistically significant differences between each condition compared to the control by the two-tailed t-test (^∗∗^p < 0.01, ^∗∗∗^p < 0.001, ^∗∗∗∗^p < 0.0001).

There are several known inhibitors of ACSL activity. Triacsin C (TC) is an analog of a polyunsaturated FA and specifically competitively inhibits the enzymes ACSL1, 3, 4 and 5 29,30,31. 2-Fluoropalmitic acid (2-FPA) is also a competitive inhibitor of ACSL activity and is an analog of palmitic acid^32^. The inhibitor rosiglitazone (RG) is an insulin-sensitizing agent that belongs to the thiazolidinediones class and also acts by reducing ACSL activity^33^. Although RG seems to be more effective on ACSL4, it also inhibits other ACSLs at higher concentrations^33^,^34^. To confirm that all three inhibitors have similar effects on ACSL activity, we performed an ACSL activity assay using a fluorescent long-chain FA substrate and analyzed the fluorescent long-chain acyl-CoA formed in the presence of ATP and CoA (see Methods). We observed that in the presence of each of the three inhibitors independently, the fluorescent acyl-CoA recovery was significantly reduced compared to the control, confirming that the three inhibitors act by reducing long-chain acyl-CoA synthesis in a dose dependent manner (Fig. 2A).

When we checked the effect of the different inhibitors on *Ct* growth, we confirmed that TC reduces *Ct* growth in a dose-dependent manner (Fig. 2B, Supplementary Fig. S4A), and that the production of infectious progeny is also significantly reduced at 56% (7.5 μM TC) (Fig. 2C) compared with the controls. We also found that 2-FPA impeded *Ct* growth in a dose-dependent manner (Fig. 2D, Supplementary Fig. S4C) and that there is a significant reduction in the formation of infectious progeny at 75% (300 μM 2-FPA) (Fig. 2E). Interestingly, this block occurs despite the accumulation of LDs in the host cell that was observed when increasing the concentrations of this inhibitor (Supplementary Fig. S4D).

Similar to the other two inhibitors, increasing concentrations of RG caused a dose-dependent block in *Ct* growth (Fig. 2F, Supplementary Fig. S4B) with a significant reduction in infectious progeny formation at 98% (100 μM RG) (Fig. 2G) compared with the controls. At low concentrations for all inhibitors with treatment from 0 to 24 hpi, we observed that only the inclusion size, but not the percentage of infected cells, was significantly reduced, indicating that the inhibitors do not affect the bacterial entry into cells (Fig. 2H). The inclusion areas were measured and compared with the inclusions from the untreated control cells. The reduction in average inclusion area was found to be 65% (7.5 μM TC), 47% (300 μM 2-FPA) and 75% (100 μM RG). The differences were statistically significant for all three inhibitors, providing further evidence that the ACSLs are important for both chlamydial growth and development.

*Ct* growth was affected by all three inhibitors regardless of the time the inhibitors were applied, although the effect was slightly more pronounced when they were added before 6 hpi (Supplementary Fig. S5A). The development of the bacteria was also affected, as shown by the infectivity assay (Supplementary Fig. S5B). We observed a significant reduction of infectious progeny for each inhibitor when added at the time of infection or at 6 hpi compared with the control. We also observed a slightly stronger but not significant effect when the inhibitors were added at earlier time points post infection.

### Host ACSLs are able to activate FAs iso-C15:0 and anteiso-C15:0 that are of *C. trachomatis* (*Ct*) origin

Similar to many other bacteria, *Ct* is able to synthetize branched-chain FAs. The most common branched-chain FAs in *Ct* phospholipids are iso-C15:0 and anteiso-C15:0 9. To assess whether host ACSLs are able to activate these *Ct* FAs that are not synthesized by human cells, we designed a competition assay with host radiolabeled FAs, palmitic (C16:0) and oleic (C18:1) acids, using an excess of the *Ct* branched-chain FAs iso-C15:0 and anteiso-C15:0 as competitors. If the competitor FA is a preferred substrate over the host radiolabeled FA, it should result in the corresponding reduction of radiolabeled acyl-CoA product. We reasoned that if host cell ACSL enzymes can activate the *Ct* branched-chain FAs, a reduction in the ability to activate substrates such as host palmitic and oleic acids would be observed in uninfected cells. Alternatively, if bacterial Acyl-CoA synthetase activity were required, competition would only be seen in infected cells.

**Figure 3:**
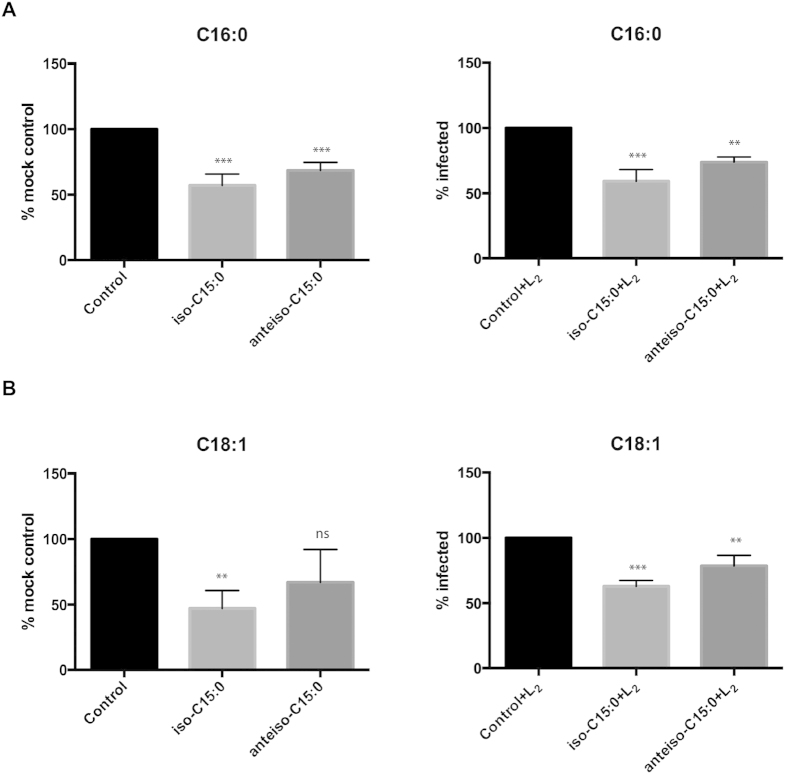
Host ACSLs activate bacterial branched-chain fatty acids (FA) iso-C15:0 and anteiso-C15:0 that are of *C. trachomatis* (*Ct*) origin. An FA competition assay was performed with uninfected and infected cells (see Methods). HeLa cells were infected (or left uninfected) with *Ct* L_2_. After 24 hpi, cells were harvested and lysed. The lysates were incubated with radiolabeled palmitic (C16:0) (**A**) or radiolabeled oleic acid (C18:1) (**B**), and an excess of the indicated competitor branched-chain FA, iso-C15:0 or anteiso-C15:0. No competitor was added to the control samples. The data were normalized to the signal of the mock or mock-infected control samples. Values (mean ± standard error for three independent experiments) are shown. The asterisks indicate statistically significant differences between each condition compared to the control by the two-tailed t- test; (ns: not significant, ^∗^p < 0.05, ^∗∗^p < 0.01, ^∗∗∗^p < 0.001).

We prepared lysates from HeLa cells, both uninfected and infected with *Ct*, and incubated them either in the presence of host radiolabeled FAs alone (controls), or with host radiolabeled FAs and *Ct* branched-chain FAs (competitors). With palmitic acid, both the uninfected and infected cells showed significantly reduced radioactivity and, therefore, reduced ACSL activity when the assay was carried out in the presence of either of the branched-chain competitors (Fig. 3A). The strongest competition was shown by the iso-C15:0 branched FA. We observed similar results when using a different host radiolabeled FA, oleic acid (Fig. 3B). In this case, although anteiso-C15:0 also reduced labeled oleic acid activation in mock-infected cells, this decrease was not statistically significant. The results, therefore, show that host ACSLs were able to activate *Ct* branched-chain fatty acids iso-C15:0 and anteiso-C15:0 in host cells (without the need for *Ct* enzymes).

### Lipid droplets (LD) are not required for *C. trachomatis* (*Ct*) entry and inclusion formation

**Figure 4:**
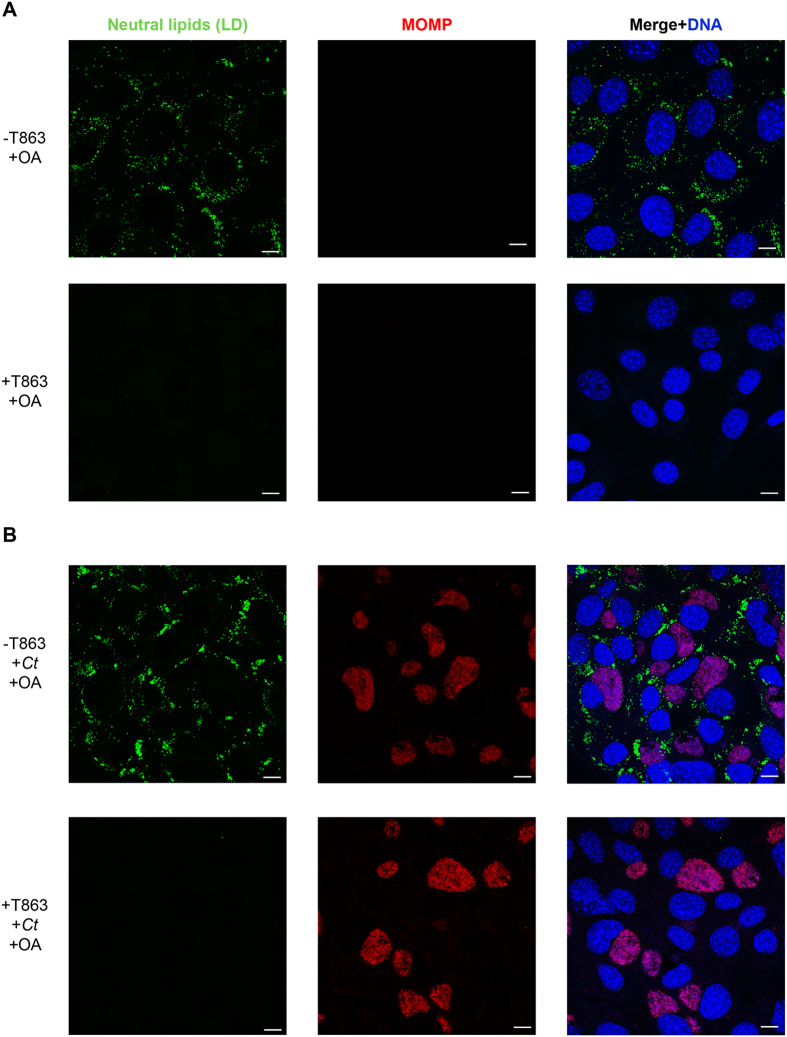
Lipid droplets (LD) are not required for *C. trachomatis* (*Ct*) entry and inclusion formation. DGAT2−/− MEF cells were treated with the inhibitor T863 or left untreated in the presence of 100 μM OA to stimulate the formation of LDs. After 16 h of incubation, cells were left uninfected (**A**) or infected with *Ct* L_2_ (**B**) without removing the inhibitor from the media. After 24 hpi, cells were fixed and labeled with anti-*Ct* MOMP antibody (red), BODIPY 493/503 for neutral lipids (green), and Hoechst for nuclear and bacterial DNA (blue). Scale bar, 10 μm.

LDs are lipid-storage organelles present in all eukaryotic cells and in some bacteria. They are not only involved in lipid homeostasis in the cells, but also participate in processes such as signal transduction, membrane and protein trafficking, and interaction with pathogens^35^. A previous publication suggested that the TC-dependent inhibition of LD formation was the reason for the block observed in *Ct* inclusion development^16^. It has also been published that RG abrogates LD formation^36^, although to our knowledge there are no previous studies of the effect of RG on *Ct* development. To determine whether the effect of these inhibitors on *Ct* growth is due to ACSL inhibition or specifically due to the abrogation of LD formation, we used an *in vitro* Mouse Embryonic Fibroblast (MEF) cell model that has a knockout in the DGAT2 enzyme and is made deficient in LD synthesis after treatment of the DGAT1 enzyme with the DGAT1-specific inhibitor T863^37^. LD synthesis occurs in the endoplasmic reticulum (ER) and involves multiple steps^38^. In the last step, diacylglycerol is converted to triacylglycerol by two enzymes: diacylglycerol O-acyltransferase 1 (DGAT1) and diacylglycerol O-acyltransferase 2 (DGAT2). When both of these enzymes are inactivated, there is no triacylglycerol synthesis and, therefore, the cells are not able to synthetize LD. When DGAT2−/− MEF cells were grown in OA-rich media and in the absence of the inhibitor T863, the cells were able to synthetize LDs (Fig. 4A, upper panels). When these cells were infected with *Ct*, the bacteria were able to grow normally (Fig. 4B, upper panels). In the presence of the inhibitor T863, there was no LD production (Fig. 4A, lower panel). Moreover, when infected with *Ct* in the absence of LD, the inclusions were seen to develop normally in these cells as observed by confocal imaging (Fig. 4B, lower panel). The same results were observed when the cells were not treated with OA, although the accumulation of LD in this case is almost nonexistent (Supplementary Fig. S6). The number of inclusions were similar with and without the presence of LDs.

### ACSLs are recruited into the *C. trachomatis* (*Ct*) inclusion in the absence of lipid droplets (LD)

**Figure 5:**
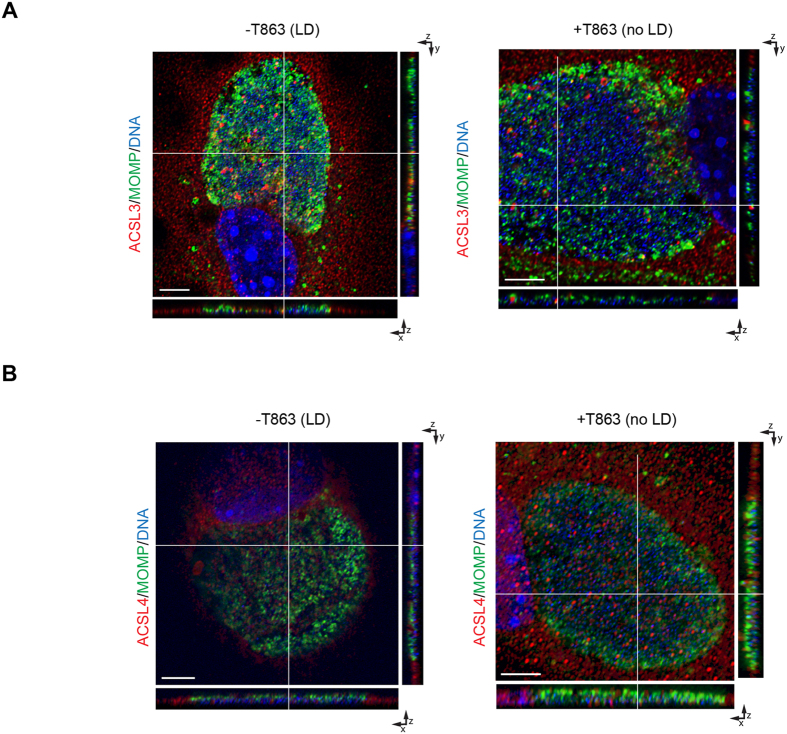
ACSLs are recruited into the *C. trachomatis* (*Ct*) inclusion in the absence of lipid droplets (LD). Control and T863 treated DGAT2−/− MEF cells were infected with *Ct* L_2_ without removing the inhibitor from the media. After 24 h, cells were fixed and labeled with anti-*Ct* MOMP antibody (green), anti-ACSL3 (**A**) or anti-ACSL4 (**B**) antibodies (red), and Hoechst to stain nuclear and bacterial DNA (blue). Representative images of z-stack projections from confocal microscopy are shown. White lines indicate localization of ACSLs within the *Ct* inclusion in the three planes, x, y, and z. Scale bar, 5 μm.

LDs are composed of a core of neutral lipids surrounded by a monolayer of phosphatidylcholine and different proteins^38^. ACSL3 and ACSL4 are two of the enzymes that are present on the LD membrane; ACSL3 is the most abundant isozyme and ACSL4 is a minor constituent^39^. ACSL3 is also present in other organelles in the cell such as mitochondria, Golgi, and the ER, while ACSL4 is present in the mitochondria, peroxisomes, and ER^40^. To determine if LDs represent the primary conduit used by ACSL3 and ACSL4 to get into the inclusion, DGAT2-/- MEF cells were treated with T863 and infected with *Ct*. By confocal microscopy, 3-D imaging of different cells corroborated that both ACSL3 (Fig. 5A) and ACSL4 (Fig. 5B) are still recruited into the *Ct* inclusion even in the absence of LDs, suggesting that other pathways are hijacked by the bacteria to recruit these enzymes.

### The block in *C. trachomatis* (*Ct*) inclusion growth by the inhibition of ACSLs is not dependent on lipid droplets (LD)

**Figure 6:**
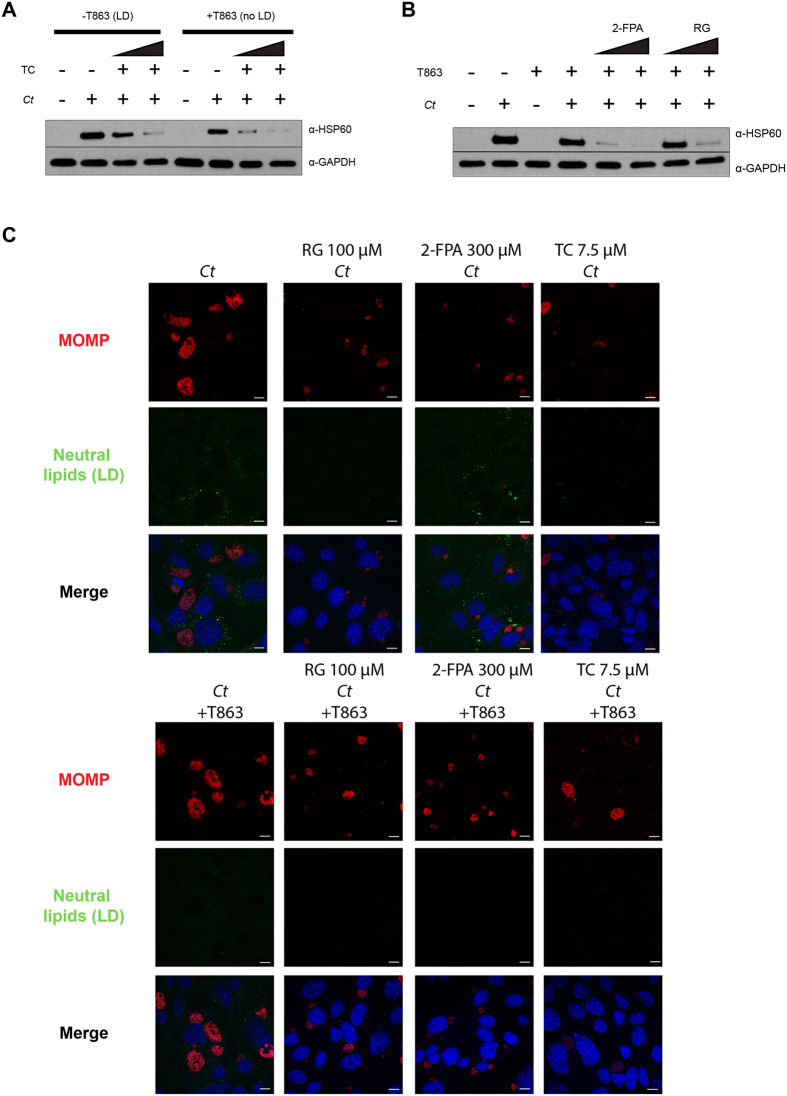
The block in *C. trachomatis* (*Ct*) inclusion growth through the inhibition of ACSLs is not dependent on lipid droplets (LD). TC (**A**), 2-FPA and RG (**B**) were added to control and T863 treated DGAT2−/− MEF cells at two different concentrations (TC: 5 μM and 7.5 μM; 2-FPA: 200 μM and 300 μM; RG: 50 μM and 100 μM) for 16 h. The cells were then infected with *Ct* L_2_ without removing the inhibitor from the media. After 24 hpi, samples were prepared for WB analysis. Membranes were probed with anti-*Ct* HSP60 antibody and anti-human GAPDH antibody as a loading control. (**C**) DGAT2−/− MEF cells were treated with the inhibitor T863 (lower panels) or left untreated (upper panels). After 8 h of incubation, the following ACSL activity inhibitors were added: 100 μM RG, 300 μM 2-FPA, and 7.5 μM TC. After 16 h of incubation, cells were infected with *Ct* L_2_, without removing the inhibitors from the media. After 24 hpi, cells were fixed and visualized by confocal microscopy. Cells were stained with anti-*Ct* MOMP antibody (red), BODIPY 493/503 for neutral lipids (green), and Hoechst for nuclear and bacterial DNA (blue). Scale bar, 10 μm.

In order to determine if *Ct* growth was still affected by the ACSL inhibitors in the absence of LD formation, we treated LD-deficient MEF cells with the ACSL inhibitors and infected them with *Ct*. *Ct* growth was still impeded in a dose-dependent manner when adding TC, indicating that the mechanism is independent of the disruption of LDs (Fig. 6A). Similarly, 2-FPA and RG also blocked *Ct* growth in the absence of LDs as shown by immunoblotting (Fig. 6B). Immunofluorescence experiments corroborated the effect of the inhibitors on *Ct* growth (Fig. 6C). These results confirm that the block in *Ct* growth occurs due to the action of the inhibitors directly on the ACSLs and not through the depletion of LDs.

### Transport of ACSLs into the *C. trachomatis* inclusion is independent of the exocytic pathway

ACSLs have different subcellular locations in the host cells. ACSL1 co-localizes with the plasma membrane, mitochondria and ER. ACSL3 is present in the mitochondria, Golgi, ER, and LDs. ACSL4 is also present in LDs as well as peroxisomes, mitochondria, and ER. ACSL5 is present in plasma membrane, ER, and mitochondria, and ACSL6 is in the plasma membrane and mitochondria^20^. Previous electron and confocal microscopy studies have shown a close association between the *Ct* inclusion and Golgi apparatus, ER and nucleus^5^,^6^,^41^,^42^,^43^,^44^. Importantly, markers for peroxisomes have been found inside the *Ct* inclusion^45^ as well as markers for LDs^17^, ER^46^, and Golgi^5^,^7^,^8^,^47^,^48^. Collectively, these observations provide several potential routes of transport for the ACSL enzymes into the *Ct* inclusion.

**Figure 7:**
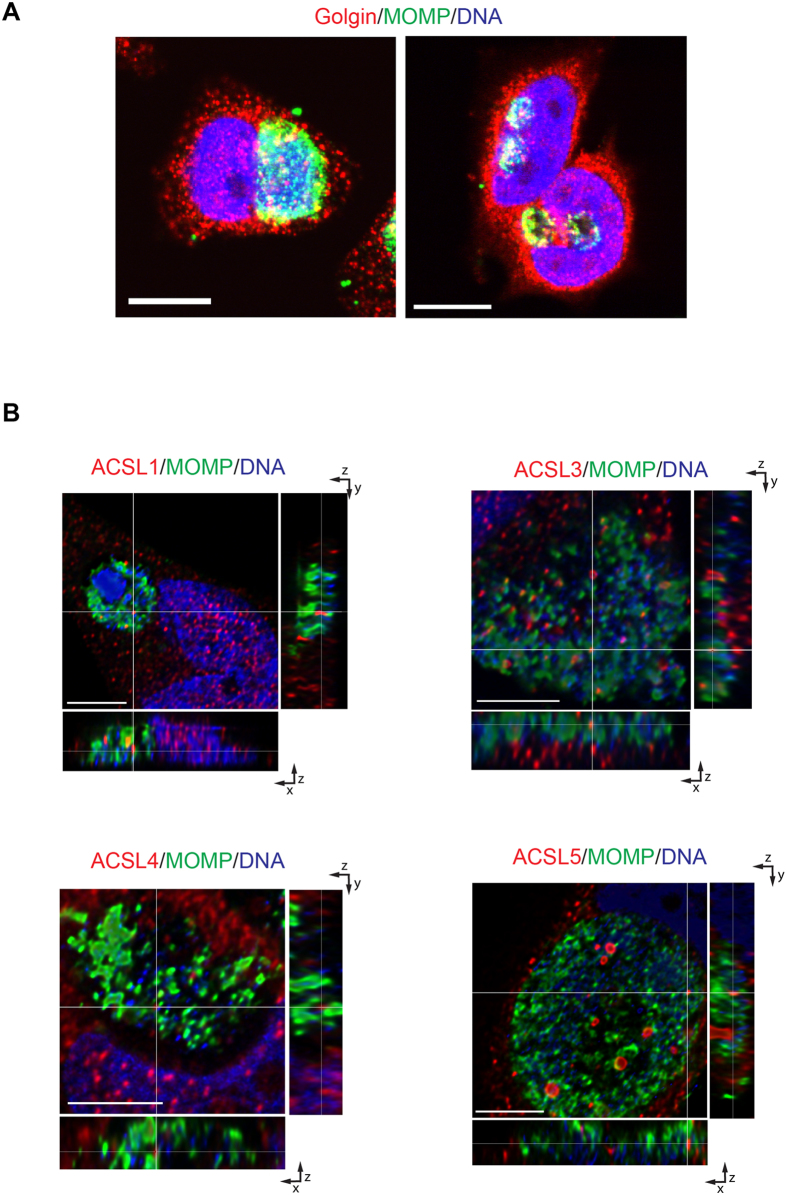
The transport of ACSLs into the *C. trachomatis* (*Ct*) inclusion is independent of the exocytic pathway. (**A**) HeLa cells were infected with *Ct* L_2_ and were either treated with Brefeldin A (BFA) from 2–4 hpi (right) or left untreated (left). After 24 hpi, cells were fixed and prepared for confocal microscopy. The inclusion was labeled with anti-*Ct* MOMP antibody (green), anti-human golgin 84 antibody (red), and Hoechst for nuclear and bacterial DNA (blue). Scale bar, 10 μm; (**B**) HeLa cells were infected with *Ct* L_2_ and treated with Brefeldin A (BFA) from 2–4 hpi. After 24 hpi, cells were fixed and prepared for confocal microscopy. The inclusion was labeled with anti- *Ct* MOMP antibody (green), anti-human ACSL-specific antibodies (red), and Hoechst for nuclear and bacterial DNA (blue). Representative images of z-stack projections from confocal microscopy are shown. White lines indicate localization of the ACSLs inside the inclusion in the three planes, x, y, and z. Scale bar, 5 μm.

To identify if the mechanism for ACSL translocation into the *Ct* inclusion was dependent on the exocytic pathway, we disrupted the pathway using chemical inhibitors. Brefeldin A (BFA) is an inhibitor of vesicular trafficking that leads to the collapse of the Golgi apparatus^49^,^50^. BFA treatment of infected cells resulted in a decrease in the inclusion size and also lack of homotypic fusion of the inclusions, as has previously been published^7^ (Fig. 7A). Under these conditions, the ACSLs are still translocated into the *Ct* inclusion, which indicates that other pathways must be required for its recruitment (Fig. 7B).

## Discussion

*Ct* is an intracellular bacterium that has undergone genome reduction during its evolution, similar to other intracellular pathogens^51^. One consequence of this is that the bacterium needs to hijack host cell pathways to acquire host metabolites and enzymes to survive. Lipids are critical for *Ct* growth and survival. Although *Ct* is able to synthetize some of the lipids it needs^52^, the organism also requires host-derived lipids such as phosphatidylcholine throughout its development^9^. Various mechanisms have been proposed to describe how *Ct* acquires phosphatidylcholine, including sphingomyelin, ceramide, cholesterol, and phosphatidic acid^4^,^6^,^7^,^8^,^53^. Apparently, different host pathways are utilized to obtain the same metabolite or metabolites. An example is the acquisition of host sphyngomyelin and cholesterol, which are recruited from multivesicular bodies, Golgi vesicles, and the ER^15^. Another example of redundancy is the use of host FAs. Even though *Ct* is able to synthetize saturated FAs on its own, the bacteria is still able to recruit and use host FAs for phospholipid synthesis^12^.

In eukaryotic cells, FAs first need to be activated by ACSLs to form acyl-CoA before they can be incorporated into phospholipids or used as a source of energy^20^. Most bacteria are able to synthesize FAs and also incorporate extracellular FAs into their membrane phospholipids. During *de novo* FA synthesis in Gram-negative bacteria, for example, an Acyl Carrier Protein, ACP, can be used to activate FA into acyl-ACP instead of acyl-CoA^54^. Additionally, some bacteria, such as *E. coli,* activate FA into acyl-CoA by utilizing their own acyl-CoA synthetase, FadD, using acyl-CoA as a precursor for phospholipid synthesis instead of acyl-ACP^55^. While *Ct* does not possess an FadD homolog, it does have an acyl-acyl carrier protein synthetase that is able to activate host FAs into acyl-ACP^12^. Since the preference of this enzyme is for saturated and straight FAs (synthetized by *Ct* or incorporated from the host), it does not explain how the 8% of OA present in *Ct* phospholipids are activated nor how bacterial branched-chain FAs are activated.

We have seen that host ACSLs are recruited to the *Ct* inclusion. However, the question remains whether the host ACSLs would be able to process branched-chain FAs of *Ct* origin. To check this, ACSL activity was measured using radiolabeled FA substrates in competition with the most common branched chain FAs produced by *Ct* (iso-C15:0 and anteiso-C15:0). Interestingly, host ACSLs were able to activate branched-chain FAs of *Ct* origin. Thus, a likely function of ACSLs in the infected cells is the activation of natural substrates such as host-cell FAs as well as bacterial FAs to form acyl-CoA. Importantly, acyl-CoA is not able to pass through lipid bilayers such as that of the *Ct* inclusion and, therefore, synthesis would need to occur inside the inclusion.

**Figure 8:**
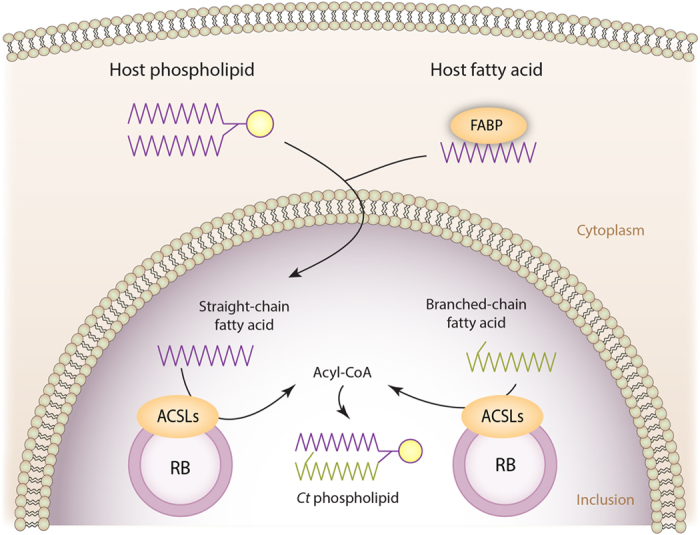
Model of fatty acid (FA) activation by *C. trachomatis* (*Ct*) using host ACSLs. *Ct* is able to recruit host-cell ACSLs and host straight-chain FAs into the chlamydial inclusion, and synthetize its own branched-chain FAs iso-C15:0 and anteiso-C15:0. FAs must be activated to acyl-CoA before they can be incorporated into phospholipids. Because acyl-CoA is not able to cross the inclusion membrane, activation must occur inside the chlamydial inclusion. We propose that host ACSL1, 3, 4, 5 and 6 are recruited into the chlamydial inclusion to activate both host straight-chain FAs and *Ct* branched-chain FAs.

The reaction catalyzed by the ACSLs requires not only FAs but also Coenzyme A (CoA) and ATP. According to the UNIPROT website, *Ct* encodes all five enzymes required in the pathway for CoA synthesis (Supplementary Data S1). It is known that *Ct* acquires ATP directly from the host as well as from bacterial glycolysis^56^. Consequently, all the metabolites necessary to activate FAs into acyl-CoA (the precursor to be incorporated into *Ct* phospholipids) would be available inside the inclusion, including the ACSLs. This model is proposed in Fig. 8.

We also found that *Ct* recruits the entire family of host ACSL enzymes into the lumen of the *Ct* inclusion early during development (Supplementary Fig. S1). These enzymes become intimately associated with the membranes of metabolically active forms of the bacteria throughout development, suggesting that they have a function in *Ct* replication (Fig. 1A,B). In addition, the ACSLs were observed in the inclusion of every infected cell that was analyzed by confocal and/or TEM. None of the ACSLs associated with the inclusion membrane and are, therefore, unlikely to be critical for inclusion membrane formation.

The reason for recruiting all five ACSLs is unknown but one possible explanation is that the bacterium utilizes different ACSLs for activating different pools of FAs. These enzymes are known to be associated with the membranes of different organelles in eukaryotic cells, and they seem to be involved in the activation and rerouting of different FAs into different lipid synthesis or degradation pathways^20^. We know that the bacterium synthetizes both saturated branched- and straight-chain FAs, and also recruits host straight-chain saturated and unsaturated FAs, so the pool of available FAs inside the *Ct* inclusion is quite diverse. It is also possible that this is another example of redundancy in the acquisition by *Ct* of host resources.

While we cannot rule out that the inhibitors we used may affect lipid homeostasis of the host and, therefore, *Ct* development, all three ACSL inhibitors, although different chemically, had a similar effect on *Ct* growth and development. It is likely that ACSL inhibition leads to a block in the synthesis of downstream FA metabolites, which could be required by *Ct.* However, the fact that the ACSLs are recruited into the inclusion early in development and that the host ACSLs are capable of processing FAs of chlamydial origin indicates a more direct role of the ACSLs in chlamydial development.

There is a growing body of work describing the interaction between host LDs and the *Ct* inclusion as well as the importance of LDs for bacterial differentiation and growth^16^,^17^. Since some of the inhibitors we used are known to have an effect on LD synthesis, we wanted to corroborate if the effect of the inhibitors on *Ct* growth was linked to the presence of LDs in the cells. We observed that, in the presence or absence of LDs, the three inhibitors were able to impair *Ct* growth (Fig. 6), which confirms that the mechanism of action of these inhibitors is independent of these organelles. Our findings do not support previous claims that the decrease in *Ct* development observed with TC treatment is due to the depletion of LDs in the cells^16^,^17^. Saka *et al.* (2015) reported that *Ct* entry into cells devoid of LDs was the same as in control cells, although the resultant infection had a reduced infectious progeny generation. We also found that, in the absence of LDs, *Ct* entry into the cells was not affected. Moreover, ACSL accumulated in the *Ct* inclusion lumen in these cells, indicating that LDs are not responsible for ACSL transport into the inclusion (Fig. 5). Our data showed that blocking ACSL activity during *Ct* infection leads to a very different phenotype (markedly reduced inclusion size) compared to the effect of depletion of LDs where inclusion formation was not affected. The role of LDs in chlamydial development appears to be more complex than previously thought and requires further research to shed light on this topic.

We determined that transport of ACSLs into the *Ct* inclusion is not only independent of LDs but also the exocytic pathway of the Golgi. It is possible that the enzymes are recruited into the inclusion via direct contact with the ER since ACSL1, 3, 4 and 5 are known to be present in this organelle, and studies have shown that ER membranes interact with the chlamydial inclusion^14^. In a previous publication, aerolysin toxin, a disruptor of the ER, was reported to affect *Ct* development^46^. Since the inclusion is affected by ER disruption, it was not possible to check for the recruitment of the ACSLs via this pathway. It is possible, though, that different pathways are utilized for the recruitment of ACSLs since they are present in several organelles, including the mitochondria, Golgi, and peroxisomes along with the ER. Consequently, the mechanism of recruitment of the ACSLs into the inclusion remains unknown and requires further study.

Taken together, our findings indicate an important role for host ACSLs in the activation of host-cell and bacterial FAs within the inclusion to promote *Ct* growth and development. In addition, it is unlikely that *Ct* requires host LDs for ACSL transport. Further studies are required to determine the pathways for ACSL recruitment into the inclusion and to analyze each of their roles in *Ct* metabolism and replication.

## Methods

### Cell culture and *C. trachomatis* infection

HeLa 229 and HEp-2 cell lines were used in experiments as described. DGAT2 −/− MEF cells were a gift from Dr. Robert V. Farese (Harvard Medical School, Boston, MA). All cell lines were grown in Dulbecco’s modified Eagle Medium (DMEM, Invitrogen, Carlsbad, CA) containing 10% heat-inactivated fetal bovine serum (Hyclone, GE Healthcare, Logan, UT) and supplemented with gentamicin (Gibco, Carlsbad, CA), vancomycin, and nystatin (Sigma Aldrich, St. Louis, MO) as previously described^57^.

*Ct* reference strain L_2_/434 was propagated in HeLa cells, harvested and purified as previously described^57^. Briefly, cells were grown to a confluence of 80% in various-sized well plates (E&K Scientific, Santa Clara, CA) with or without coverslips (Electron Microscopy Sciences, Inc., Hatfield, PA) and infected with *Ct* at an MOI of 1 or 50 depending on the experiment, for 1–2 h (30 minutes shaking at room temperature, and 1 h 30 minutes at 37 °C). Fresh media (same as above) was added and cells were incubated further until the time of analysis.

### Lipid droplet (LD) formation and abrogation

LD formation was induced with 100 μM OA (Sigma-Aldrich). The abrogation of LD formation was performed using MEF cells with a knockout in DGAT2, one of the enzymes involved in LD synthesis. These cells are still able to synthetize LDs because of the presence of the enzyme DGAT1. T863 (Sigma-Aldrich) is a specific inhibitor of the enzyme DGAT1, and when used at 20 μM for 16 h on the DGAT2−/− knockout MEF cells, no LD formation is observed^37^.

### Confocal microscopy

HeLa, HEp-2, and DGAT2 −/− MEF cells were grown and infected when appropriate on 12-mm glass coverslips (Electron Microscopy Sciences) in 24 well plates (E&K Scientific). Cells were fixed with 4% formaldehyde in PBS for 20 minutes, permeabilized with 0.02% of saponin, and incubated with the following primary antibodies: mouse anti-human ACSL1 (Abnova, Taipei, Taiwan), mouse anti-human ACSL3, mouse anti-human ACSL4, mouse anti-human ACSL5 (Santa Cruz Biotechnologies, Dallas, TX), rabbit anti-human ACSL6, rabbit anti*-Ct* MOMP (Virostat, Portland, ME), and mouse anti-*Ct* LPS (Virostat). Cells were then incubated with the appropriate Alexa Fluor secondary antibodies (Life Technologies, Carlsbad, CA) using the manufacturer’s recommended dilutions. Samples were incubated with BODIPY 493/503 (Life Technologies) to detect neutral lipids and LDs. Nuclei and bacterial DNA were detected using Hoechst 33258 (Life Technologies). Images were collected on a Zeiss LSM710 and LSM510 confocal inverted microscope. Image processing was performed using the Huygens Essential (Scientific Volume Imaging, The Netherlands) and Imaris (Bitplane AG, Switzerland) software programs.

For intensity distribution profiles, traces of the intensity of the signal (y axis) for ACSLs (red), *Ct* (green) and DNA (blue) were plotted as a function of the distance in μm (x axis) of the section of the cell indicated by a red arrow to quantify the results.

### Transmission Electron Microscopy (TEM)

HEp-2 cells were grown and infected on sapphire disks coated with carbon. The cells were prefixed in a media containing 4% formaldehyde and 0.2% glutaraldehyde (Electron Microscopy Sciences), and high pressure frozen in either a Bal-Tec HPM 010 or Leica HMP 100 high-pressure freezer and freeze-substituted in 1% osmium tetroxide and 0.1% uranyl acetate in acetone^58^. Freeze-substitution was performed in a Leica AFS2 device by raising the temperature from −90 °C in increments of 2 °C/h up to −25 °C followed by increments of 5 °C/h up to 0 °C^59^. Infiltration in Lowicryl HM20 resin was carried out using different concentrations of a mix of acetone-resin or pure resin, and polymerization was carried out for 2 hours in an oven at 100 °C. Sections of 70 nm thickness were carved from the polymerized blocks of cells using a microtome (Ultracut E; Reichert-Jung, Depew, NY) and deposited on hexagonal copper grids at a mesh size of 100 lines/inch. The grids were post-stained with 2% aqueous uranyl acetate in 70% methanol for 4 minutes and lead citrate for 2 minutes as previously described^60^. The sections were labeled with the same primary antibodies previously used in the confocal microscopy section; mouse anti-*Ct* HSP60 (Santa Cruz Biotechnologies) and mouse anti-human Cytokeratin 18 (Abcam, Cambridge, MA); and secondary antibodies conjugated to 12 nm or 18 nm gold particles (Jackson Immunoresearch, West Grove, PA) for 1 h at RT. Images were collected on a Tecnai T12 transmission electron microscope (FEI Inc., Hillsboro, OR) operating at 120 kV (Electron Microscopy Lab, UC Berkeley, Berkeley, CA), and Philips/FEI BioTwin CM120 TEM and Hitachi 7600 TEM (Institute for Basic Biomedical Sciences Microscope Facility, Johns Hopkins University School of Medicine, Baltimore).

### Acyl-CoA synthetase activity measurement

An ACSL-modified assay was used as previously described^26^,^61^. Briefly, HeLa cells were grown in 6-well plates (E&K Scientific), harvested, washed 3 times with cold PBS and resuspended in STE buffer (8.5% sucrose, 10 mM Tris-HCl pH 8, 0,5 mM EDTA) supplemented with protease inhibitors Pepstatin A, Leupeptin and PMSF (Sigma-Aldrich). The cells were lysed by several freeze-thaw cycles, and total protein content was quantified using the DC Protein Assay according to the manufacturer’s instructions (BioRad, Hercules, CA). 60 μg of protein from each sample were incubated with 0.075 μl of 2 mM FA BODIPY 500/510 C_4_, C_9_ (Life Technologies) solubilized in α-cyclodextrin, 40 mM Tris-HCl pH 7.5, 10 mM ATP, 10 mM MgCl_2,_ 0.2 mM CoA and 0.2 mM DTT (all from Sigma-Aldrich). Duplicate reactions were incubated at 37 °C for 20 min, and the reaction was stopped using Dole’s solution (isopropanol:heptane:2N H_2_SO_4_ 40:10:1) (Sigma-Aldrich). The newly synthesized fluorescent acyl-CoA collects in the aqueous phase and, after several extractions with heptane (to remove the unprocessed FAs in the organic phase), the fluorescent signal was measured using a Tecan Infinite M200 PRO (Tecan Group Ltd., Männedorf, Switzerland).

### Fatty acid competition assays

HeLa cells were grown in 6-well plates (E&K Scientific) and the next day infected with *Ct* L_2_ as previously described. At 24 hpi, cells were harvested by trypsinization, washed 3 times with cold PBS and resuspended in STE buffer supplemented with protease inhibitors. The cells were lysed by several freeze-thaw cycles, and total protein content was quantified using the method of Lowry *et al.*^62^. Benzene solutions of radiolabeled human FAs, [1-^14^C]oleic acid (C18:1, Moravek Biochemicals, Brea, CA) or [1-^14^C]palmitic acid (C16:0, Moravek Biochemicals), were evaporated to dryness under a stream of nitrogen in the absence or presence of competing branched-chain FAs, bacterial iso-C15:0 or anteiso-C15:0 (Larodan, Limhamn, Sweden), and solubilized in a mixture of α- plus methyl-β-cyclodextrins (Sigma-Aldrich; each 10 mg/ml in 10 mM Tris, pH 8.0). Radiolabeled FAs were used at a final concentration of 20 μM and competing FAs at a final concentration of 60 μM. Duplicate HeLa cell suspensions (5 μg protein for C18:1 and 15 μg protein for C16:0) were incubated with solubilized FAs in 40 mM Tris-HCl pH 7.5, 8.5 mM ATP, 8.5 mM MgCl_2,_ 0.12 mM CoA and 0.12 mM DTT (all from Sigma-Aldrich) for 20 min at 37 °C. The reactions were terminated by the addition of Dole’s solution, unreacted substrate was removed by heptane extraction, and radioactivity in the aqueous phase (acyl-CoA product) was quantitated by liquid scintillation counting as previously described^61^. After correction for blanks (samples without cell lysates added), enzymatic activity was expressed as nanomoles acyl-CoA formed in 20 minutes per mg protein. The data are shown as percentage of the enzymatic activity from control mock-infected cells or control-infected, depending on the experiment, incubated without any competitor fatty acid.

### Inhibition of *C. trachomatis* growth

HeLa cells were grown in 24-well plates and treated with different concentrations of the inhibitors TC, 2-FPA and RG (Santa Cruz Biotechnologies) either before or after infection, as indicated in each particular experiment. For TC, 5, 7.5, 10, 15, and 25 μM were used depending on the experiment. For 2-FPA, 25, 50, 100, 150, 200, 250, 300, 350 μM were used depending on the experiment. For RG, 10, 25, 50, 100, 150 μM were used depending on the experiment. For the experiments with Brefeldin A (BFA) (Sigma Aldrich), 10 μM was used and added from 2–4 hpi. Cells were infected with *Ct* as described previously, and processed either for confocal microscopy or for WB.

### Western Blot immunodetection

Cells were grown in 12-well plates, and infected with *Ct* as described. After 24 h, cells were lysed with Laemmli buffer with β-mercaptoethanol (Bio-Rad). Samples were boiled for 10 minutes and loaded on pre-cast SDS-PAGE gels (Bio-Rad). After transfer, PVDF membranes (Life Technologies) were blocked for 1 hour in 5% non-fat milk Tris Buffered Saline supplemented with Tween 20 (TBST) buffer (Quality Biological, Gaithersburg, MD) and probed with different antibodies. Primary antibodies including anti-*Ct* HSP60 (Santa Cruz Biotechnologies) and GAPDH (EMD Millipore, Billerica, MA,), and peroxidase-conjugated secondary antibodies (Santa Cruz Biotechnologies) were diluted in blocking buffer and incubated for 1 h at RT. Detection was performed with ECL WB Detection Reagents (GE Healthcare, Lafayette, CO).

### Quantitation of infectious progeny

To quantify the production of infectious progeny following treatment with inhibitors, infected HeLa cells were scraped into the fresh media as stated above, lysed with ddH_2_0, and 10-fold serial dilutions were used to infect fresh HeLa monolayers that had been previously plated for 24 h. Inclusions were visualized by immunofluorescence using a rabbit anti*-Ct* MOMP antibody (Virostat). The inclusion forming units (IFUs) were expressed as IFU/mL.

### Quantitation of chlamydial inclusion size

Cells were stained with a rabbit anti*-Ct* MOMP antibody (Virostat) and imaged using confocal microscopy, as described previously. The images were analyzed using Imaris X64 software to identify Regions of Interest (ROI) coinciding with the inclusions. The software was used to calculate the mean inclusion area (in μm^2^) based on the ROIs.

### siRNA transfection and ACSL antibody specificity

siRNAs against human ACSL1 (ON-TARGET plus™ SMARTpool^®^, #L-011654-00, GE Healthcare Dharmacon, Lafayette, CO), human ACSL3 (custom-designed siRNA, Sense: 5′-UGUUUAUUCUGGCCUAUAAUU-3′ and Antisense: 5′- UUAUAGGCCAGAAUAAACAUU-3′, Dharmacon), human ACSL4 (Silencer^®^ Select Validated siRNA, #4390824, ThermoFisher Scientific, Foster City, CA), human ACSL5 (ON-TARGET plus™ SMARTpool^®^, #L-006327-01, Dharmacon) and human ACSL6 (ON-TARGET plus™ SMARTpool^®^, L-007748-00, Dharmacon) were independently used to transfect HeLa cells using Lipofectamine^®^ 2000 (Invitrogen) according to the manufacturer’s instructions. Briefly, 12 pmol siRNA was diluted in 200 μl Opti-MEM^®^ I Medium without serum (Invitrogen) in a 12-well plate. Two μl Lipofectamine^®^ 2000 was added to each well containing the diluted siRNA, mixed gently and incubated for 20 min at RT. 100,000 HeLa cells (in suspension after trypsinization) were added to each well, gently mixed and incubated for 48 h at 37°C in 5% CO_2_. Cells were either infected with *Ct* for 24 h or left uninfected. Cells were processed for confocal microscopy and for WB as described above.

### Statistical analysis

Statistical analysis was performed using the software program GraphPad Prism (GraphPad Software, La Jolla, CA). Statistical significance between groups was determined by the two-tailed Student’s t-test. A *p* value of less than 0.05 was considered to be statistically significant. Data are shown as the mean ± standard error of *n* independent experiments.

## Additional Information

**How to cite this article**: Recuero-Checa, M. A. *et al.* *Chlamydia trachomatis *growth and development requires the activity of host Long-chain Acyl-CoA Synthetases (ACSLs). *Sci. Rep.* **6**, 23148; doi: 10.1038/srep23148 (2016).

## Supplementary Material

Supplementary Information
