# Supplementary material for: Chlamydia trachomatis growth and development requires the activity of host Long-chain Acyl-CoA Synthetases (ACSLs)
Source: Sci Rep. 2016 Mar 18;6:23148. doi: 10.1038/srep23148 (PMC4796813; doi:10.1038/srep23148)
Supplement: Supplementary Information [file srep23148-s1.pdf]

Supplementary Information

Chlamydia trachomatis **growth and development requires the activity of host Long-chain Acyl-CoA Synthetases (ACSLs)**

Maria A. Recuero-Checa[1,2,+], Manu Sharma[1,+], Constance Lau[1], Paul A. Watkins[3,4], Charlotte A. Gaydos[2], Deborah Dean[1,5*]

[1]Center for Immunobiology and Vaccine Development, UCSF Benioff Children's Hospital Oakland Research Institute, Oakland, CA, 94609, USA

[2]Department of Infectious Disease, Johns Hopkins University, Baltimore, MD, 21205, USA

[3]Hugo W. Moser Research Institute at Kennedy Krieger, Baltimore, MD, 21205, USA

[4]Department of Neurology, Johns Hopkins University School of Medicine, Baltimore, MD, 21205, USA

[5]Department of Bioengineering, University of California at Berkeley and San Francisco, CA, USA

[+]These authors contributed equally to the research

*Corresponding author:

Deborah Dean, MD, MPH

ddean@chori.org

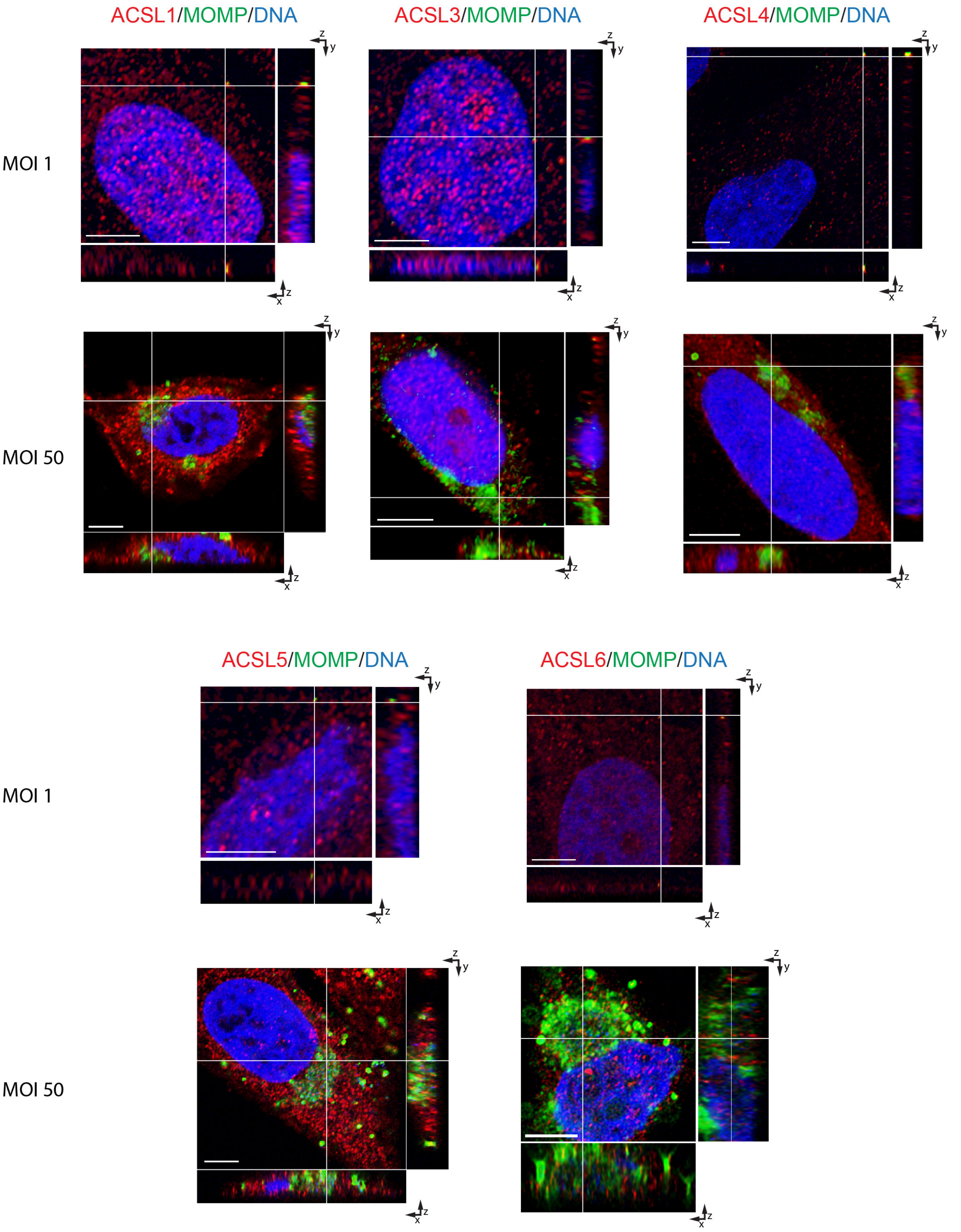
ACSL1/MOMP/DNA
ACSL3/MOMP/DNA
ACSL4/MOMP/DNA
MOI 1
MOI 50
ACSL5/MOMP/DNA
ACSL6/MOMP/DNA
MOI 1
MOI 50
z
y
x
z

Supplementary Figure S1. ACSLs are translocated into the *C. trachomatis (Ct)* inclusion during early stages of infection. HeLa cells were infected with *Ct* L2 for 6 h at a MOI of 1 (upper panel) and at a MOI of 50 (lower panel). Cells were fixed and prepared for confocal microscopy. The inclusion was labeled with anti-chlamydial MOMP antibodies (green), ACSL-specific antibodies (red), and Hoechst for nuclear and bacterial DNA (blue). Representative images of z-stack projections from confocal microscopy are shown. White lines indicate localization of ACSLs within the *Ct* inclusion in the three planes, x, y, and z. Scale bar, 5 µm.

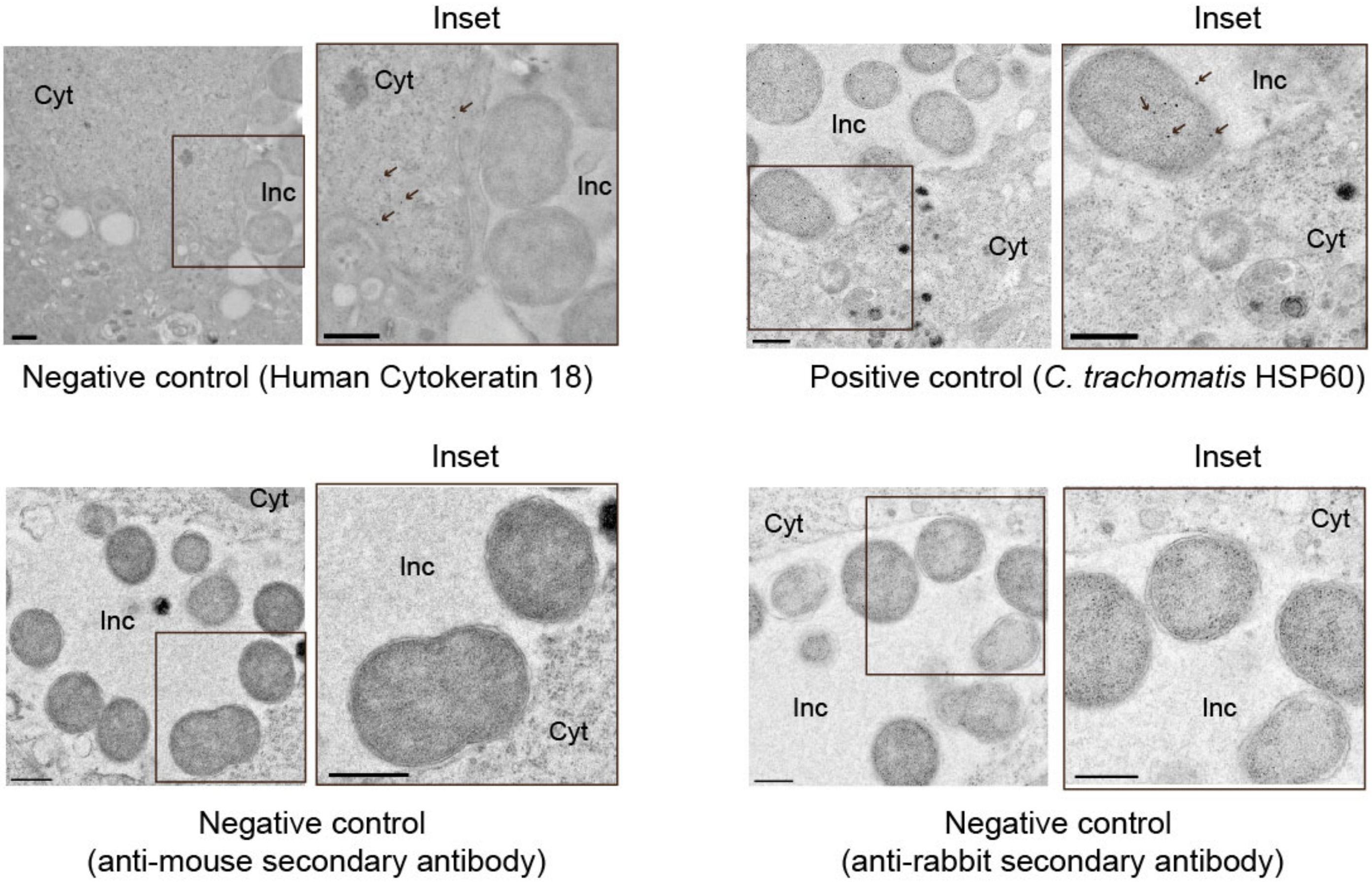

Supplementary Figure S2. Negative controls for transmission electron microscopy. HEp2 cells were infected with *Ct* L2 for 24 h at an MOI of 1, and prepared for TEM. Proteins were labeled with specific antibodies conjugated to 12 or 18 nm gold particles. Human cytokeratin 18 was used as a negative control (top left panel) as it localizes to the host-cell cytoplasm. *Ct* HSP60 was used as a positive control (top right panel), as it is found within the inclusion lumen. Secondary antibody controls verified the absence of non-specific signal (bottom left and right panels). Arrows indicate immunogold labeling of respective proteins. Inc, inclusion; Cyt, cytoplasm. Scale bar, 500 nm.

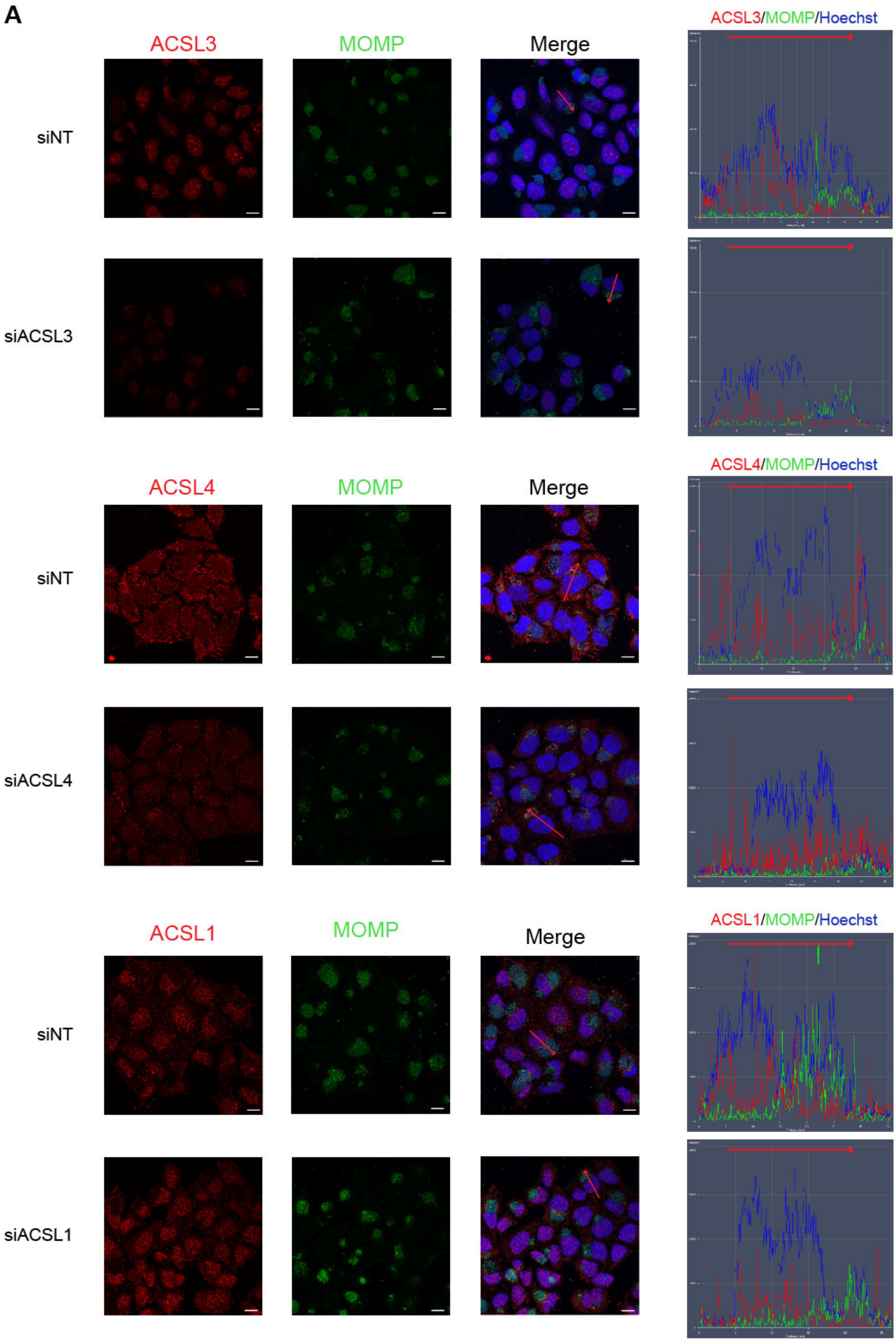

A
ACSL3
MOMP
Merge
ACSL3/MOMP/Hoechst
siNT
siACSL3
ACSL4
MOMP
Merge
ACSL4/MOMP/Hoechst
siNT
siACSL4
ACSL1
MOMP
Merge
ACSL1/MOMP/Hoechst
siNT
siACSL1

B

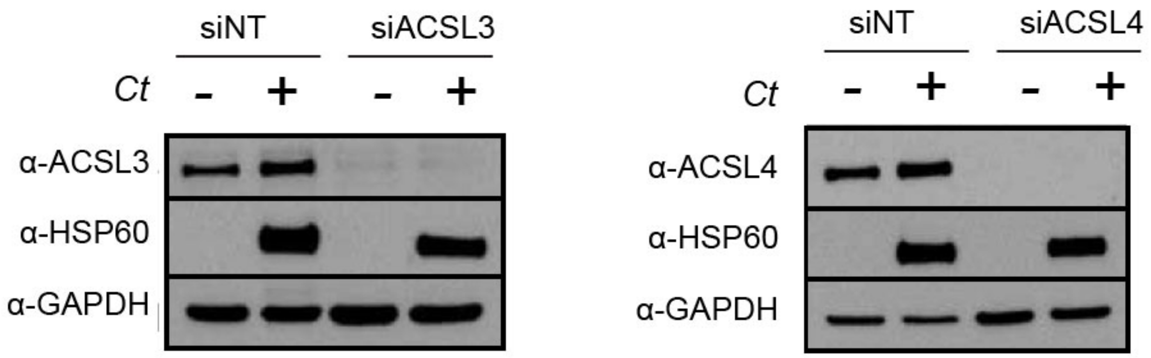

Supplementary Figure S3. Specificity of ACSL antibodies. HeLa cells were transfected with the corresponding ACSL siRNA for 48 h, and then infected with *Ct* L2 for 24 h or left uninfected (see Methods). A) Confocal microscopy with intensity distribution profiles confirmed the specificity of the ACSL antibodies. The inclusion was labeled with anti-*Ct* MOMP antibody (green), ACSL-specific antibodies (red), and Hoechst for nuclear and bacterial DNA (blue). Scale bar, 10µm. Traces of the intensity of the signal (y axis) for the ACSLs (red), *Ct* (green) and DNA (blue) were plotted as a function of the distance in µm (x axis) of the section of the cell indicated by a red arrow. The intensity distribution profiles for the corresponding confocal images show a decrease in fluorescence in the cytoplasm and within the inclusion when ACSLs are knocked down, demonstrating the specificity of the ACSL antibody. B) Western blot analysis confirmed the siRNA knock down and the specificity of the ACSL antibodies. Membranes were probed with the indicated anti-human ACSL antibody, anti-chlamydial HSP60 and anti-human GAPDH antibody as a loading control.

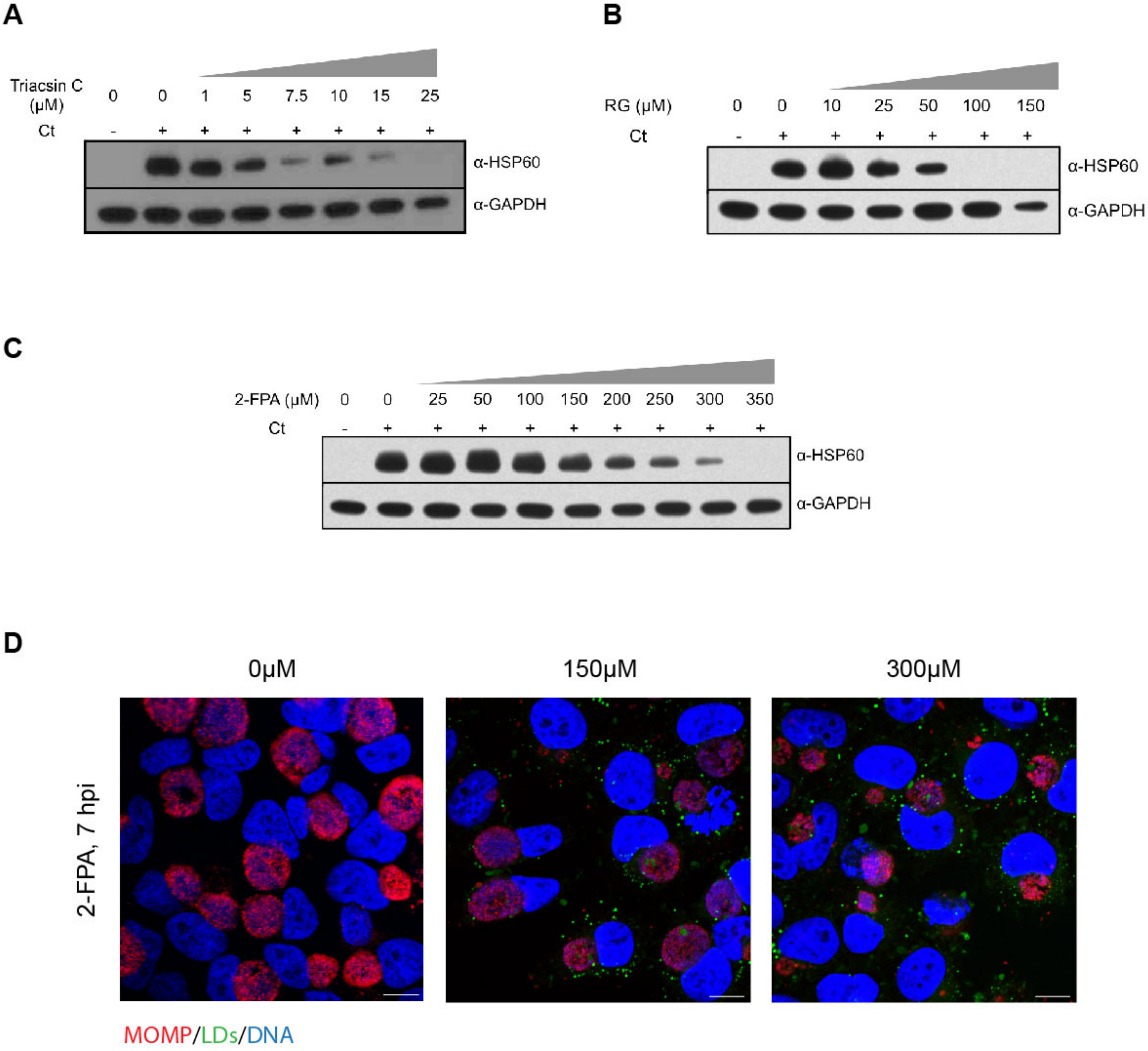

Supplementary Figure S4. ACSLs are required for *C. trachomatis (Ct)* inclusion growth. A) HeLa cells were treated with the inhibitor TC at the indicated concentrations for 16 h and then infected with *Ct* L2 without removing the inhibitor from the media. At 24 hpi, the cells were fixed and prepared for Western blot analysis. Membranes were probed with anti-*Ct* HSP60 antibody and anti-human GAPDH antibody as loading control. The same experiment was carried out with the inhibitor RG (B) and 2-FPA (C). D) HeLa cells accumulate LD in presence of the inhibitor 2-FPA. Cells were infected with *Ct* and treated with 2-FPA at the indicated concentrations from 7 to 24 hpi or left untreated. At 24 hpi, the cells were fixed and prepared for confocal microscopy. They were labeled with anti-*Ct* MOMP antibody (red), BODIPY 493/503 for LD (green) and Hoechst for nuclear and bacterial DNA (blue). Scale bar, 10µm.

A

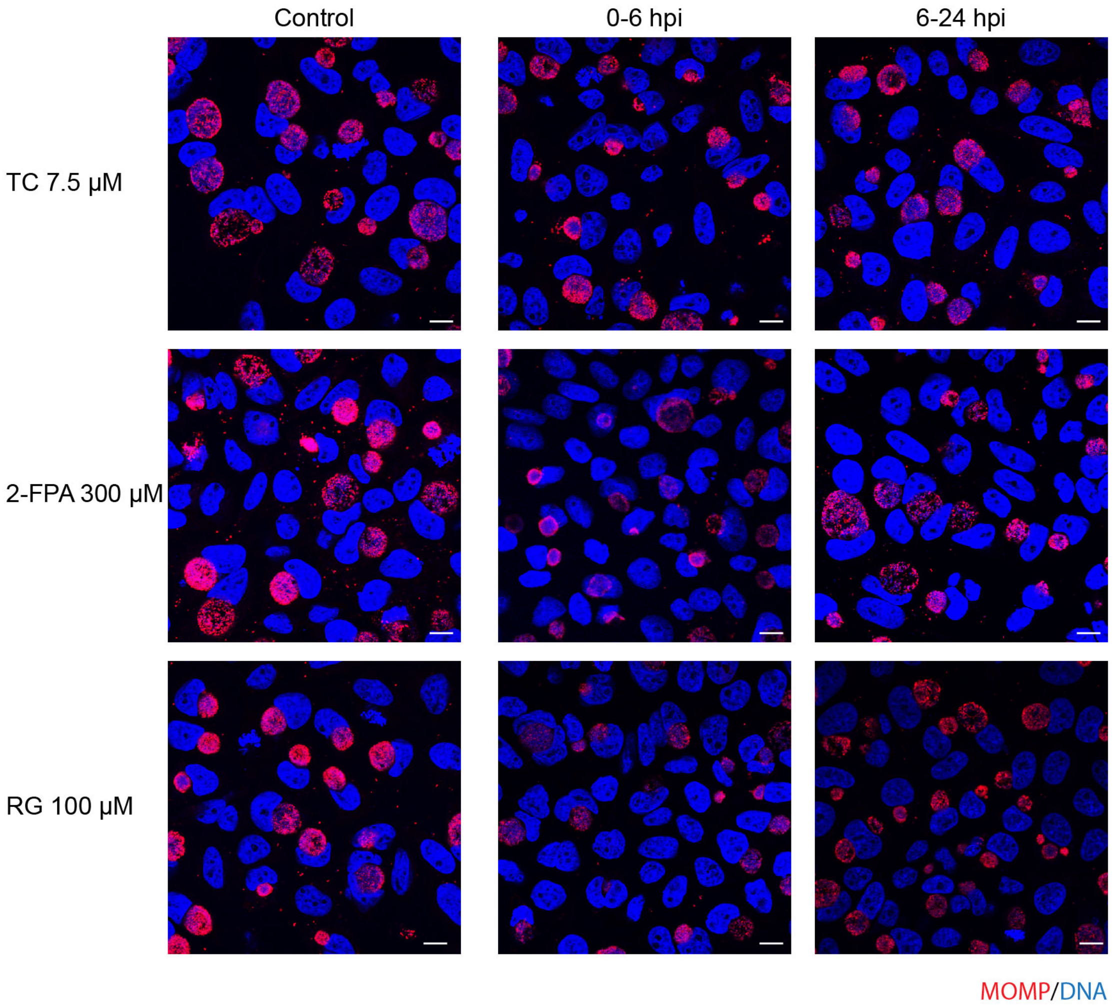

Control
0-6 hpi
6-24 hpi
TC 7.5 μM
2-FPA 300 μM
RG 100 μM
MOMP/DNA

B

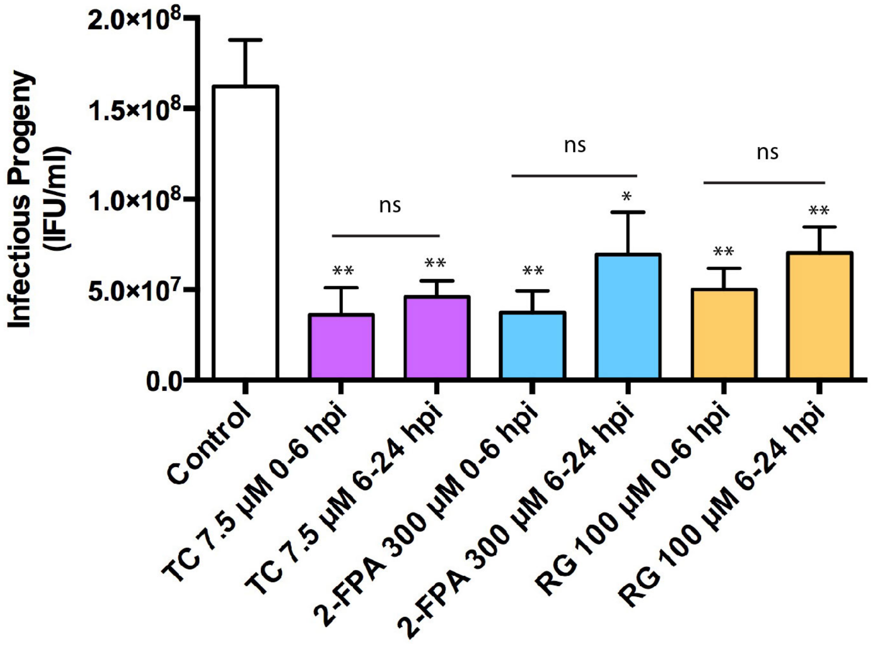

Infectious Progeny (IFU/ml)
2.0×10^8
1.5×10^8
1.0×10^8
5.0×10^7
0.0
ns
ns
ns
**
**
**
*
**
**
Control
TC 7.5 μM 0-6 hpi
TC 7.5 μM 6-24 hpi
2-FPA 300 μM 0-6 hpi
2-FPA 300 μM 6-24 hpi
RG 100 μM 0-6 hpi
RG 100 μM 6-24 hpi

Supplementary Figure S5. Inhibitors of ACSLs impede *C. trachomatis (Ct)* growth during and after inclusion formation. A) HeLa cells were left untreated or treated with the inhibitors TC, 2-FPA or RG at the indicated concentrations either from 0-6 hpi infection or from 6-24 hpi with *Ct* L2. At 24 hpi, cells were fixed and prepared for confocal microscopy. The inclusion was labeled with anti-*Ct* MOMP antibody (red), and nuclear and bacterial DNA were labeled with Hoechst (blue). Scale bar, 10 µm. B) HeLa cells were treated with the three ACSL inhibitors at the indicated concentrations from 0-6 hpi or 6-24hpi and infected with *Ct* L2. The cultures were used for reinfecting new HeLa cell monolayers and analyzed for infectivity and production of progeny. Values (mean ± standard error for three independent experiments) are shown as inclusion forming units (IFU)/mL. The asterisks indicate statistically significant differences between each condition compared to the control by the two-tailed t-test (*$p<0.05$, **$p<0.01$). The horizontal bars represent comparisons between the two concentrations used for each inhibitor by the two-tailed t-test (ns: non-significant differences).

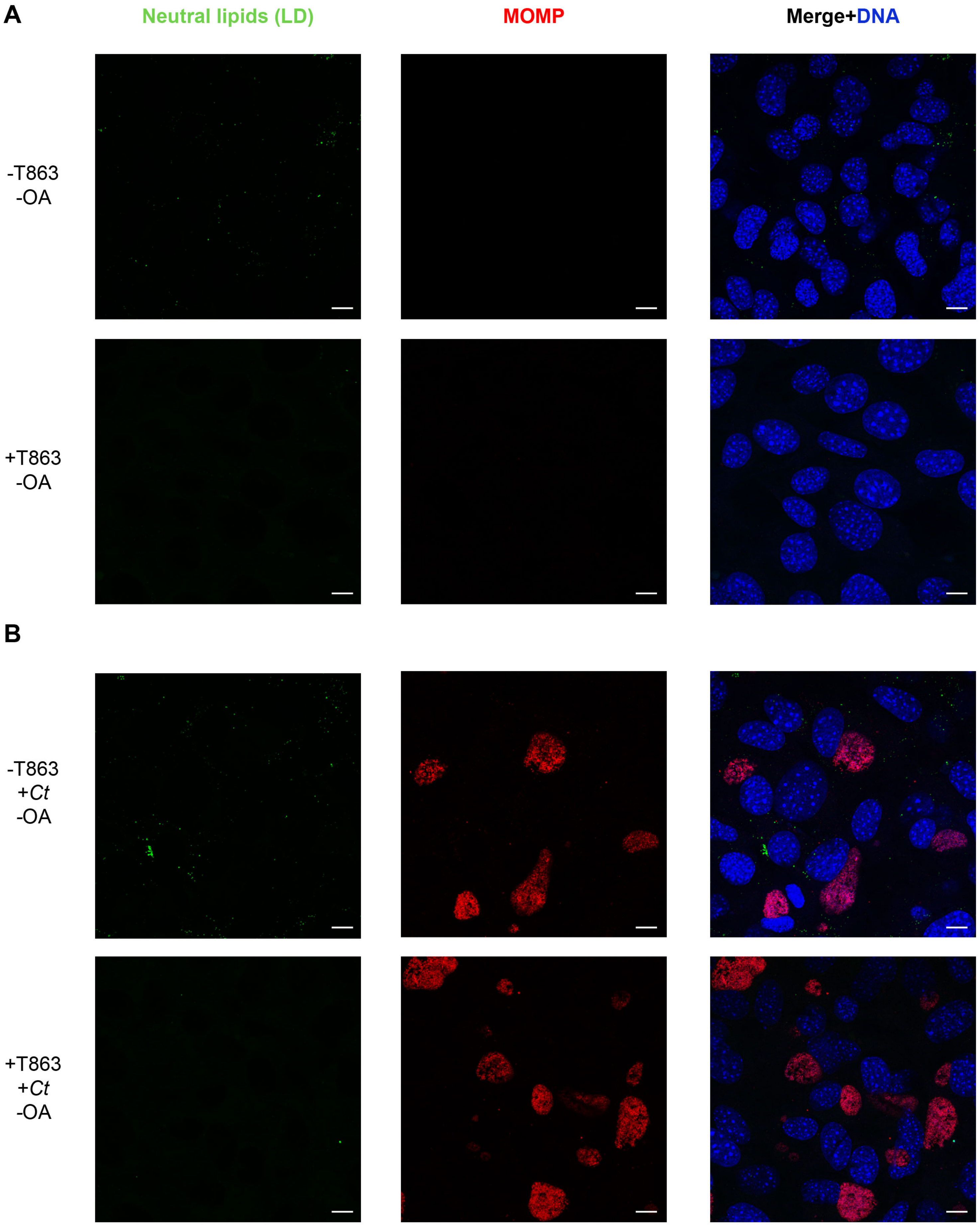
A
Neutral lipids (LD)
MOMP
Merge+DNA
-T863
-OA
+T863
-OA
B
-T863
+Ct
-OA
+T863
+Ct
-OA

Supplementary Figure S6. Lipid droplets (LD) do not accumulate naturally in DGAT 2-/- MEF cells. DGAT2-/- MEF cells were treated with the inhibitor T863 or left untreated in the absence of OA. Cells were infected with *Ct* L2 (B) or left uninfected (A), without removing the inhibitor from the media. After 24 hpi, cells were fixed and labeled with anti-*Ct* MOMP antibody (red), BODIPY 493/503 for neutral lipids (green), and Hoechst for nuclear and bacterial DNA (blue). Scale bar, 10 µm.

A)

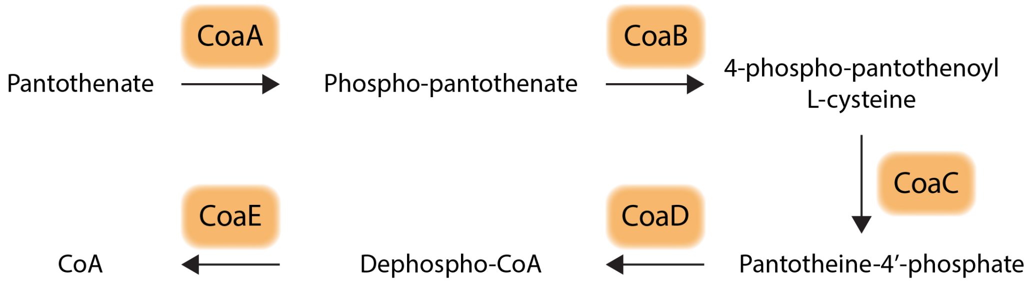

B)

>tr|A0A0E9GCV6|A0A0E9GCV6_CHLTH Pantothenate kinase OS=Chlamydia trachomatis GN=**coaA**_2 PE=3 SV=1
MTLPFSAAEQAHSPISPSYTHRSTPLSTFMPISSTQWRALAATTELPLTDKDVRALAALG
DPIGIAEADAIYRPLSALMQMYARHTGQLMSESHSFIGLPEHRTPWIIGIAGSVAVGKST
AARLLRELMRRWPQTPHVDLVTTDGFLYPNATLTKHGLMDRKGFPESYDRHALLNFLSAV
KSGVGNLHVPVYDHISYDIVPGKWITVDNPDVLIVEGLNVLQPPRMSSSDGEFRAVSDYF
DFSIYLDAPEDALEQWYMDRFRNLRKTAFTNTNSFFRSYANLSDEEADNTARRLWRTINL
PNLRENVAPTRSRATIILTKGADHSVETIYLRKL

>tr|A0A0E9G7P6|A0A0E9G7P6_CHLTH DNA/pantothenate metabolism flavoprotein OS=Chlamydia trachomatis GN=**coaBC**_1 PE=4 SV=1
MNSFDTYPSQHKLEDISHSHRTIVVGVTAGIAAYKIPQLIRELRACRYTVNVVPTRASLD
MVGAVTWEAISANPVYCDVSDNAADVTHVRIGESADLFVIAPATANTIAKLAHGIADNLL
TATALVVKCPVVIVPAMHTQMWEHPATQANIATLKNRGVIVLGPECGRLTGSDSGMGRMK
EPHDIAAACISILEGNDFFEASEDGSNPHSSDTPSRDSSLSDRKDMQGVRYAISAGGTHE
PIDPVRFIGNKSTGMMGIALANEAAQRGAAVTLVGAHLASDVLASVDPRIHYCSVETACE
LHEAMNNLAAETDVIIMSAAVADYRPERVASEKLKKTEGTLESLRLVQNPDILSELAHHR
RRSNQIVVGFAAETGSVEHTALEYGILKAQRKGADLMIVNQVGDNIGFGNVATSVTILDS
AGKCLAAAHGTKPDVAHTIIDTISSVIRGRSASPAATVQEKND

>tr|A0A0E9EEU1|A0A0E9EEU1_CHLTH Phosphopantetheine adenylyltransferase OS=Chlamydia trachomatis GN=**coaD**_2 PE=3 SV=1
MNTEISHVVVPGTFDPITLGHLDVIRRARKMFPQVSVAVALSARKNETGTTFSLEHRVEM
VRASLDEAHLSDVNVYPFEGLLVQFVKSIGAQAVVKGLRAMTDFEYELQQSDLNTRMNPD
IESIYVMSNPKYGFISSSVVREIASMGADVSMMVPSCVLQHLYNK

>tr|A0A0E9DRR7|A0A0E9DRR7_CHLTH Dephospho-CoA kinase OS=Chlamydia trachomatis GN=**coaE**_1 PE=3 SV=1
MTQIHMGGASADLLRPFVIPTDRALLVGLSGGIGSGKSSVATAFASLGAVVADADAIARE
VVEPGSVGLRRIEEVFGDAILNDDGTLNRGALAQKVFTDASARKTLEAITHPLIAERSRQ
ILSTAQPGNIALYDVPLLTEQHMHNQFDAVMMVDVPLDIRLSRLQARGMTIDEARKRIAS
QANSDERRAICHIWITNTGSLVDLQRLVATVHAQWLTNSGTTGSPTGR

C) http://www.uniprot.org/

Supplementary Data 1. Coenzyme A synthesis pathway in *C. trachomatis (Ct)*. A) Coenzyme A synthesis pathway in *C. trachomatis*. B) FASTA sequences of the enzymes involved in the pathway. C) URL link to the Uniprot website from where the sequences were obtained.
